# Supplementary material for: Glycation of Host Proteins Increases Pathogenic Potential of Porphyromonas gingivalis
Source: Int J Mol Sci. 2021 Nov 8;22(21):12084. doi: 10.3390/ijms222112084 (PMC8585099; doi:10.3390/ijms222112084)
Supplement: Supplementary file 1 [file ijms-22-12084-s001.zip › ijms-1439281-supplementary.pdf]

Supplementary Materials

Glycation of host proteins increases pathogenic potential of *Porphyromonas gingivalis*

Michał Śmiga [1], John W. Smalley [2], Paulina Ślęzak [1], Jason L. Brown [2,†], Klaudia Siemińska [1], Rosalind E. Jenkins [3], Edwin A. Yates [4] and Teresa Olczak [1,*]

[1] Laboratory of Medical Biology, Faculty of Biotechnology, University of Wrocław, 14A F. Joliot-Curie St., 50-383 Wrocław, Poland; michal.smiga@uwr.edu.pl (M.Ś.); paulina.stepien2@uwr.edu.pl (P.Ś.); klaudia.sieminska@uwr.edu.pl (K.S.)
[2] Institute of Life Course and Medical Sciences, School of Dentistry, The University of Liverpool, Pembroke Place, Liverpool L3 5PS, UK; e-mail josmall@liv.ac.uk
[3] CDSS Bioanalytical Facility, Department of Pharmacology and Therapeutics, Institute of Systems, Molecular and Integrative Biology, Faculty of Health and Life Science, The University of Liverpool, Liverpool, L69 3GE, UK; R.Jenkins@liv.ac.uk
[4] Institute of Systems, Molecular and Integrative Biology, Faculty of Health and Life Science, The University of Liverpool, Liverpool, L69 7ZB, UK; e.a.yates@liv.ac.uk
† Present address: Oral Sciences Research Group, Glasgow Dental School, School of Medicine, College of Medical, Veterinary and Life Sciences, University of Glasgow, Glasgow, G2 3JZ , UK
* Correspondence: teresa.olczak@uwr.edu.pl (T.O.)

Table S1 . Degree of glycation of haemoglobin as measured by assay for 5-hydroxymethylfurfural (5-HMF). Values are expressed as the molar ratio of 5-HMF to haemoglobin subunit. Assays were carried out on three separate preparations of both glycated and un-glycated hemoglobin samples.

Sample number	Molar ratio (5-HMF:hemoglobin)	
	Glycated hemoglobin	Un-glycated hemoglobin
1	1.183	0.210
2	1.171	0.201
3	1.295	0.200
Average (mean±SD)	1.216±0.068	0.203±0.005

Table S2. Comparison of hemoglobin glycation sites.

Lysine residue	Glycation sites detected in this study	*In vivo* and *in vitro* glycation sites [1]	*In vivo* glycated sites [2]	*In vitro* glycated sites [3]	*In vitro* glycated sites [4]
7(α)	-	+	+	-	-
11(α)	-	-	-	-	-
16(α)	+	+	+	+	+
40(α)	+	+	+	-	-
56(α)	+	-	+	-	+
60(α)/61(α)	+	+	-	+	-
90(α)	+	-	-	-	-
99(α)	+	-	-	-	-
127(α)	+	-	+	-	-
139(α)	+	-	+	+	-
8(β)	+	+	+	-	+
17(β)	+	+	+	+	-
59(β)	+	-	-	+	-
61(β)	-	-	+	-	-
65(β)/66(β)	+	+	+	+	-
82(β)	+	-	-	+	+
95(β)	+	-	-	+	-
120(β)	+	+	-	+	-
132(β)	+	-	+	+	-
144(β)	+	+	+	+	+

1. Shapiro, R.; McManus, M.J.; Zalut, C.; Bunn, H.F. Sites of nonenzymatic glycosylation of human hemoglobin A. *J Biol Chem* **1980**, *255*, 3120-3127.
2. Zhang, X.; Medzihradszky, K.F.; Cunningham, J.; Lee, P.D.; Rognerud, C.L.; Ou, C.N.; Harmatz, P.; Witkowska, H.E. Characterization of glycated hemoglobin in diabetic patients: usefulness of electrospray mass spectrometry in monitoring the extent and distribution of glycation. *J Chromatogr B Biomed Sci Appl* **2001**, *759*, 1-15.
3. Delpierre, G.; Vertommen, D.; Communi, D.; Rider, M.H; Van Schaftingen, E. Identification of fructosamine residues deglycated by fructosamine-3-kinase in human hemoglobin. *J Biol Chem* **2004**, *279*, 27613-27620.
4. Ito, S.; Nakahari, T.; Yamamoto, D. The structural feature surrounding glycated lysine residues in human hemoglobin. *Biomed Res* **2011**, *32*, 217-223.

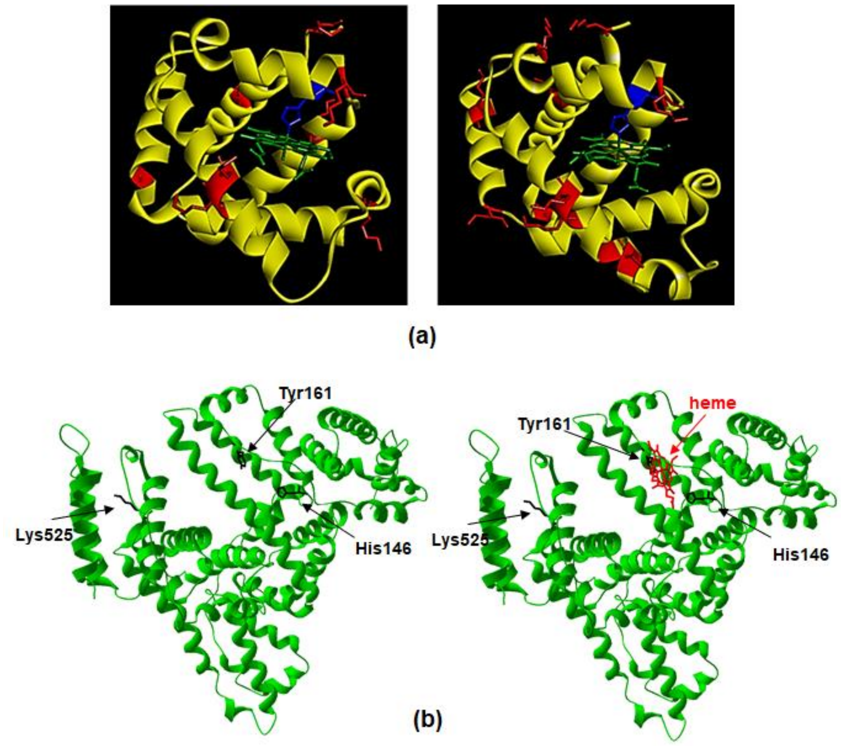

Figure S1. Main glycation targets in hemoglobin and albumin. (**a**) Glycated lysine residues in the α-chain (left panel) and β-chain (right panel) of horse methemoglobin are shown. The protein structure was constructed using the structure deposited in the PDB data base (PDB ID: 2MHB). Glycated lysine residues (red), the heme molecule (green), and proximal histidine residues (His87 in α-chain and His92 in β-chain) (blue) are indicated. (**b**) Location of heme-binding site (red) with amino acids coordinating heme iron (Tyr161 and His146; black) and main glycated lysine residue (Lys525) in human serum albumin are indicated.

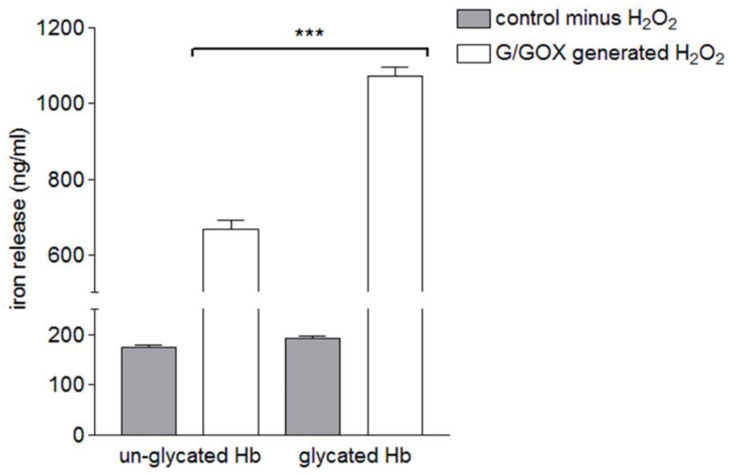

Figure S2. Quantification of iron (III) release from glycated and un-glycated hemoglobin. Glycated and un-glycated hemoglobin (200 μM with respect to tetramer) were exposed to a continuous flux of H_2O_2 generated by glucose/glucose oxidase (G/GOX). ***P<0.001

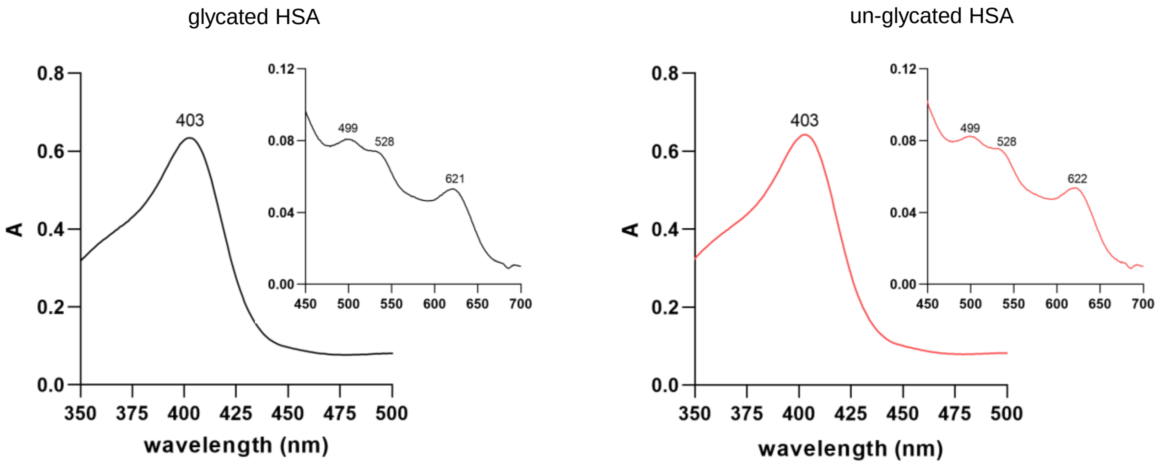

Figure S3. **Characterization of glycated human serum albumin**. The UV-visible spectra of the glycated (left panel) and un-glycated albumin (right panel) are shown. A, absorbance.

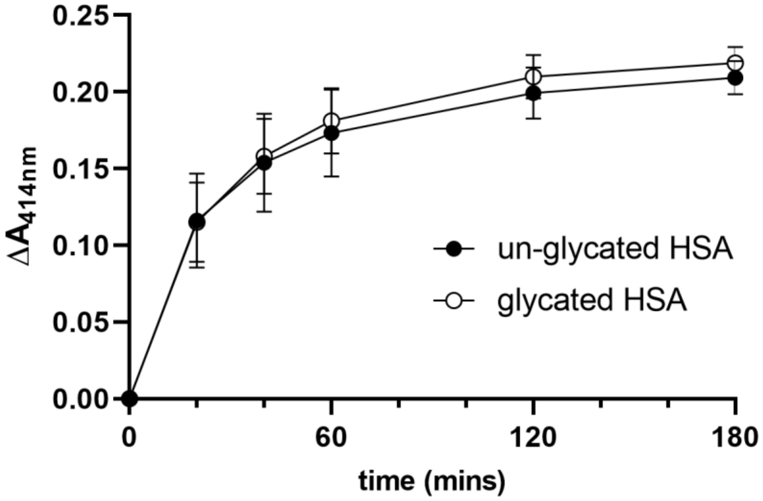

Figure S4. Comparison of HmuY-Fe(III)heme complex formation from glycated and un-glycated albumin. 5 μM human serum albumin (HSA) was incubated with an equimolar concentration of HmuY. Absorbance values derived from difference spectra at 414 nm were used to create the curve demonstrating HmuY-Fe(III)heme complex formation. ΔA, difference absorbance.
